# Evaluation of TIMS-derived peptide mobility measurements: calibration strategy and MS1/MS2 discrepancies

**DOI:** 10.1007/s00216-026-06633-9

**Published:** 2026-06-26

**Authors:** Anh Tran, Tytus Mak, Meghan Burke Harris

**Affiliations:** Mass Spectrometry Data Center, Biomolecular Measurement Division, National Institute of Standards and Technology (NIST), Gaithersburg, MD 20899 USA

**Keywords:** Ion mobility, Proteomics, Mass spectrometry

## Abstract

The widespread adoption of trapped ion mobility coupled to mass spectrometry for proteome analysis necessitates high-quality data measurement of both ion mobility and mass spectra. In this study, the accuracy of ion mobility measurements in the context of mass spectral (MS) library building and searching is evaluated. Key factors influencing measurement accuracy, such as calibration strategy and the timing of acquisition relative to the chromatographic apex, are systematically investigated. A calibration system based on a synthetic peptide mixture is shown to improve calibration performance. Further, reproducibility of MS1- and MS2-associated mobility measurements in data-dependent acquisition (DDA) is analyzed. Overall, this work identifies methods to improve ion mobility measurement accuracy for proteomics analyses using a standardized synthetic peptide mixture.

**Graphical abstract:**

**Figure:**
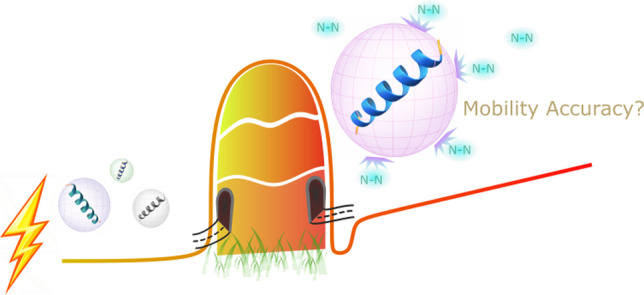

**Supplementary Information:**

The online version contains supplementary material available at 10.1007/s00216-026-06633-9.

## Introduction

Separation and identification metrics vary across different LC–MS-based analytical techniques. In liquid chromatography (LC), analytical separation is widely utilized, but identification based on retention time (RT) typically requires nearly similar experimental setups and predictions to be reliable [1–3]. In tandem mass spectrometry (MS/MS), MS2 is commonly used as the primary identification metric, while MS1 in quadrupole instruments mainly serves for separation or isolation of ions [4–6]. Ion mobility spectrometry (IMS) is a powerful technique for resolving and filtering analytes by size, shape, and charge [7–10]. Recently, trapped ion mobility spectrometry (TIMS) has become increasingly popular in proteomic experiments due to its high duty cycle, rapid separation, and fast sampling rate [7, 8, 11]. By adding an extra dimension to conventional LC–MS workflows, IMS not only provides a post-ionization separation but can also produce physiochemical metrics that can be used to corroborate identifications, especially when chromatographic and mass separation are insufficient [12–15]. IMS-based analytical technology is an excellent approach to enhance the confidence of peptide identifications and is compatible with, as well as complementary to, LC and high-frequency mass analyzers [16, 17].

The integration of CCS values into proteomic workflows as a metric to enhance peptide identification confidence requires accurate and reproducible CCS measurements [18–26]. To ensure reliability, careful validation of IMS measurements is accomplished using drift tube (DT) IMS, regarded as the gold standard for CCS determination and a benchmark for evaluating newer IMS technologies [27–31]. Because CCS is a normalized physicochemical property of an ion-gas pair presenting its rotationally averaged momentum transfer as they interact and is generally more reproducible than elution voltages across various instrument-specific parameters, it is possible to harmonize data across different ion mobility platforms.

Here, the ion mobility measurement accuracy of consensus spectrum data generated from in-house software, IsoPique, was evaluated. While reproducibility is critical for consistent measurements, it is beyond the scope of this paper. Although many CCS calibration strategies exist that apply broadly across molecule classes [32, 33], our approach emphasizes a tailored strategy: using calibrants with similar shape, charge, and size. This approach is used with traveling wave ion mobility calibration systems [34–37], and here, we will show that the approach is also beneficial for TIMS-based proteomics experiments. Given the unique properties of peptide ions (such as their multiply charged states and helical structures [38, 39]), an appropriate calibrant system that closely mimics these characteristics is essential for accurate measurements [37, 40–42]. The calibrant system used here, NIST Reference Material (RM) 8321 [43], is a synthetic mixture of approximately 440 peptides chosen from tryptic peptides frequently observed in human proteomic analysis. This mixture covers a broad dynamic range and diverse physicochemical properties, making it well suited not only for calibration but also as a quality control standard for LC–MS peptide analyses [44]. TIMS-TOF measurement results were compared with CCS values obtained from drift tube ion mobility spectrometry (DT-IMS).

Finally, parameters including ramp time, accumulation time, and exclusion window were evaluated for their impact on ion mobility measurement accuracy during MSMS acquisition, referred to as MS2-associated mobility. During the process of DDA Parallel Accumulation Serial Fragmentation (PASEF) analysis, the precursor and product ions’ mobility is analyzed in a multiplexed manner, and measured values are compared to match the correct product ions to corresponding precursor ions [8, 11]. Recommendations from this analysis improve the alignment of MS1- and MS2-associated ion mobility measurements.

## Methods

### Samples

NIST RM 8321 peptide mixture was diluted fourfold in 0.1% formic acid and 2% acetonitrile solution (v/v). Under current experimental conditions (microflow LC), 5 µL of a 4 × diluted RM 8321 solution was injected per run. At this amount, a single vial can support up to 40 injections, corresponding to ~ 120 injections per unit (three vials). The total number of uses will depend on the chromatographic configuration; for example, nanoflow LC workflows would further increase the number of injections per vial due to lower sample consumption. ESI-L LCMS Tuning Solution (Agilent) was used for IM calibration, for both DT-IM and TIMS runs. LC–MS grade Water and ACN (Honeywell) solvents were used for chromatographic runs and sample preparation.

### LC conditions

The peptide mixtures were separated via reverse-phase separation on a low microflow LC. Full gradient information is available in SI Fig. 5. ACQUITY UPLC 1 mm × 100 mm Peptide CSH C18 (130 Å, 1.7 µm) was used, and column temperature was set at 40 °C.

### TIMS

Gas flow was adjusted, and mobility was calibrated to Agilent Tune mix daily. VIP-HESI (Bruker) source was used, with source parameters based on Moritz et al [45]. Full details of source, TIMS, and MS parameters can be found in SI Fig. 6.

### DT-IM

The NIST RM 8321 peptide mixture was separated with the same LC conditions mentioned above, on an Agilent 6560 A DT-IM QTOF with Agilent 1290 UHPLC. DT electronics were set to 1274 V DT entrance voltage and 224 V DT exit voltage. See SI Fig. 7 for the full details on other acquisition parameters. Ultra-high purity (UHP) nitrogen was used as the input gas in all experiments. The gas supply was further purified via a filtration system (Part Number: RMSN-4 Big Universal Trap for nitrogen).

The data was acquired in 4-bit multiplexing mode and demultiplexed with PNNL preprocessor. Then, features were extracted in Agilent MassHunter IM-MS Browser Version 10.0.1, with a default peptide profile setting, and were exported and analyzed in Python. The feature list was exported. CCS values were calculated based on the Mason-Schamp equation (taking into consideration the CCS calibration). CCS calibration was performed with the Agilent Tune mix (the same one used for the TIMS-TOF). More information can be found in SI Fig. 7 and SI Table 1.

### Clustering of features and peptide ID workflow

Features of the DDA IM-MS analysis from multiple replicates were consolidated into clusters and then identified via library searching. This is further explained below.

The LC IM-MS results were analyzed using an in-house developed data analysis package, IsoPique, to cluster features of technical replicates and search them against NIST peptide mass spectral libraries to provide peptide spectral matches. For a given precursor *m*/*z* associated with an MS2 spectrum, its corresponding MS1 chromatographic trace is extracted (and considered the “primary” XIC), as well as all potential isotopic traces associated with it (“ancillary” XICs). The primary XIC undergoes validation via non-linear least squares Gaussian fitting, and ancillary XICs are validated via peak shape similarity to the primary XIC utilizing Spearman’s rank correlation coefficient. This validated set of MS1 XICs is then used to elucidate the charge and monoisotopic *m*/*z* (essentially building a validated isotopic envelope), which are used to annotate the original MS2 spectrum. These data are used to cluster MS2 spectra across multiple chromatograms, with any discrepancies in the charge state and monoisotopic *m*/*z* values resolved through a consensus algorithm. This results in data clusters of unique spectral analytes which contain MS2 and MS1 data across all chromatograms. These clusters may contain MS2 spectra with different precursor *m*/*z* values, but have been determined to be isotopes of the same analyte. If a data cluster contains more than one MS2 spectrum, a consensus spectrum is generated to represent the cluster, resulting in a single MS2 spectrum representing each data cluster. This set of representative MS2 spectra is then searched against the NIST human tryptic peptide library via NIST MSPepSearch [46].

Results were further processed and visualized in Python.

### Data curation and alignment of MS1- and MS2-associated IMS values

During the DT-IMS data analysis workflow, gas-phase conformers exhibiting complex gas-phase conformers were excluded during DT-IMS CCS data curation. For the remaining species, the most intense peak was selected as the representative mobility value. If alternative chromatographic methods or gradients are employed, users would need to regenerate the calibration curve to reflect the discrepancies in solvent composition during elution.

After peptide identification, the corresponding chromatogram peak apex and MS1 mobility values were extracted (calculated from the consolidated feature via IsoPique). For each consensus MS2 event (represented by the points on the plot), its retention time was compared to the associated chromatographic peak apex. Likewise, the mobility value of each consensus MS2 event was compared with the corresponding MS1 mobility. In cases where multiple mobility conformers were present, the conformer with the highest-intensity peak was selected. These two discrepancies are then plotted on 2D plots, color-coded by the charge of the original peptide analyte (Fig. 5).

## Results/discussion

### Peptide-specific ion mobility calibration strategy

**Fig. 1:**
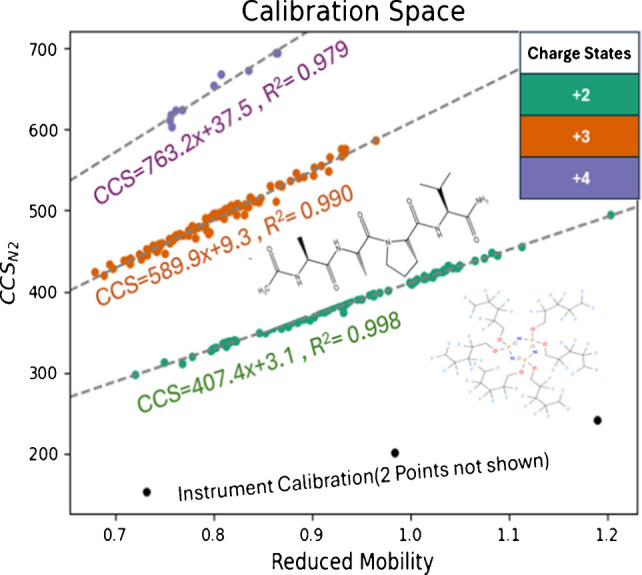
The calibration space and linear regression of RM 8321 peptides, compared to the commercial calibration mixture. The *x*-axis represents the analyte’s reduced mobility (1/*K*_0_), the *y*-axis represents the CCS N_2_ values (from DT-IMS measurements) while the colors indicate the charge state

For peptide ions, their helical linear structures with multiple charge states stand in contrast to the globular and singly charged species in the fluoro-phosphazene calibration mixture that is widely used for trapped ion mobility calibration. With an aim of selecting calibrants of similar shape and charge states as tryptic peptide ions and a lack of available calibration mixtures specific to peptide IM measurements, a ground truth peptide mixture (NIST RM 8321) was assessed. To construct the calibration curve, consensus spectra from three technical replicates were collected, filtering out mobility values that were not shared across the replicates. Then, peptides with low MS signal intensity and complex separation behavior were filtered out (i.e., multimodal distributions), leading to mostly peptides with mobility values that have ≈2.5% difference from DT measured CCS N_2_ values. The resulting linear regressions have a strong fit and are distinctly positioned in the calibration space (Fig. 1).

**Fig. 2:**
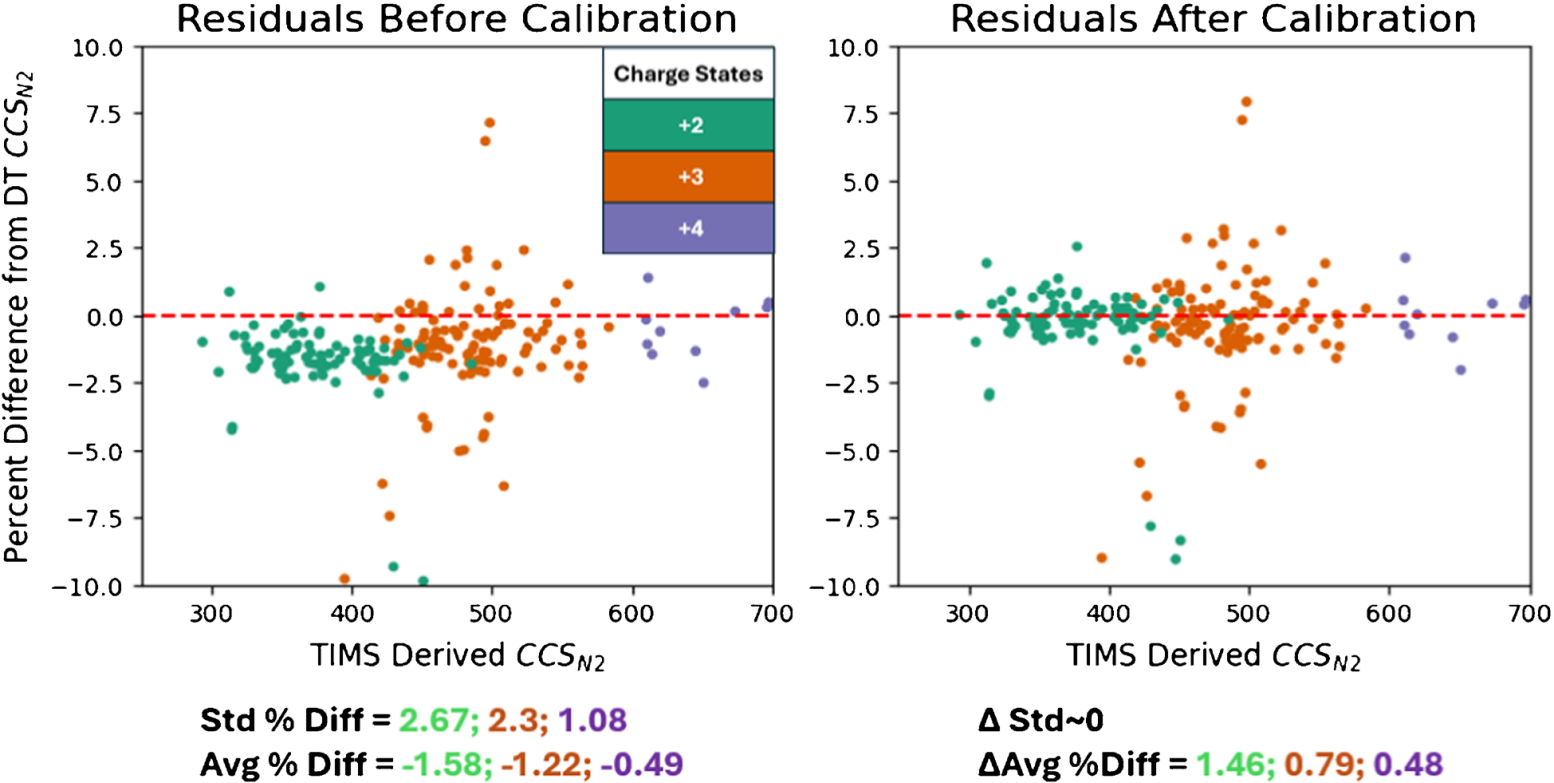
A comparison between TIMS-derived CCS N_2_ values and DT-derived CCSN_2_ values of RM 8321 peptides following calibration using the 3 linear regressions derived from the RM 8321 calibrant system. After calibration, the delta Avg % difference is the change in average percent difference from DT CCS from before calibration with RM 8321

The RM 8321 peptides were calibrated by applying the appropriate regression equations, and their results were compared against DT CCS N_2_ values (Fig. 2). The change in average % difference is most pronounced in + 2 charge states and becomes progressively less significant in higher charge states, such as + 4. These trends highlight the importance of applying charge state–specific fitting approaches. Accuracy in mobility alignment is particularly crucial when distinguishing between peptides with similar mobilities. For example, coeluting species with overlapping isotopic envelopes can benefit significantly from an accurate mobility metric for confident identification (a visual explains this further in SI Fig. 1). This improved accuracy is crucial for building reliable libraries and for users generating data intended for searches against those libraries. The lowest *R*^2^ values observed for + 4 charge state peptides likely result from their more complex behavior during mobility separation and fewer data points across the regression, characterized by narrower mobility ranges and fewer data points for reliable sampling. As a preliminary observation, peptide CCS values derived from TIMS do not consistently agree with those derived from DT-IMS, further necessitating the proposed calibration strategy.

### Discrepancies between MS1- and MS2-associated reduced mobilities

So far, the focus has been on MS1 IM values. However, the accuracy of IM measurements can be further modulated by the inclusion of MS2-associated mobility measurements from DDA PASEF analyses. Alignment between MS2- and MS1-associated mobility values will be tested to ensure compatibility. High discrepancy may result in compromised quality of MS2 fragmentation spectra and incorrect assignment of mobility value to the analyte of interest. Although DDA PASEF acquisition was tested here, the underlying principles can have implications in PRM and data-independent acquisition (DIA) PASEF modes.

**Fig. 3:**
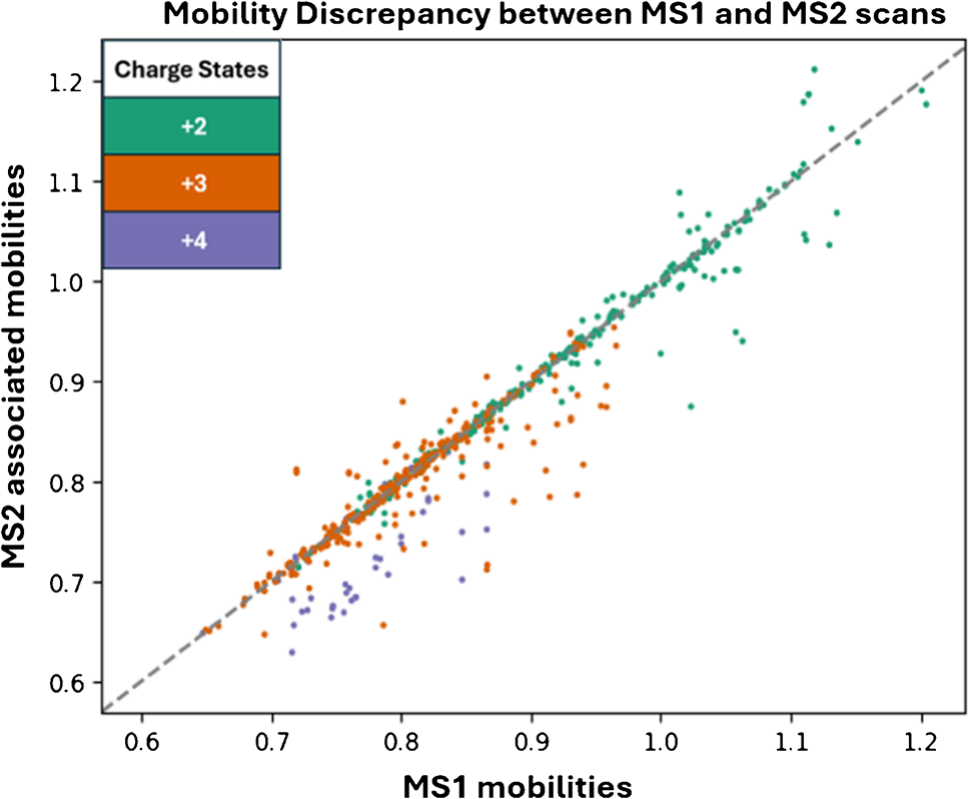
Correlation between MS1 and MS2 associated mobility measurements across three charge states. MS1 mobilities (*x*-axis) are derived from consensus MS1 spectra, while MS2-associated mobilities (*y*-axis) are based on the consensus MS2 spectra. The dashed line represents perfect agreement (1:1) between the two measurements, with residuals plotted around the line. Data were acquired using a 100 ms ramp time and a 15 s exclusion window

Due to the stochastic nature of data-dependent acquisition (DDA), MS2 acquisition times are not precisely controlled, which can lead to discrepancies between the assigned MS2-associated mobility values and the corresponding MS1 mobility values for a given peak. While the MS1 mobility value is derived from the aggregation of frames across the entire chromatographic profile, individual MS2-associated mobility values may differ, especially when acquired from low-intensity signals at the tail ends of eluting peaks. This conflict is illustrated in Fig. 3.

**Fig. 4:**
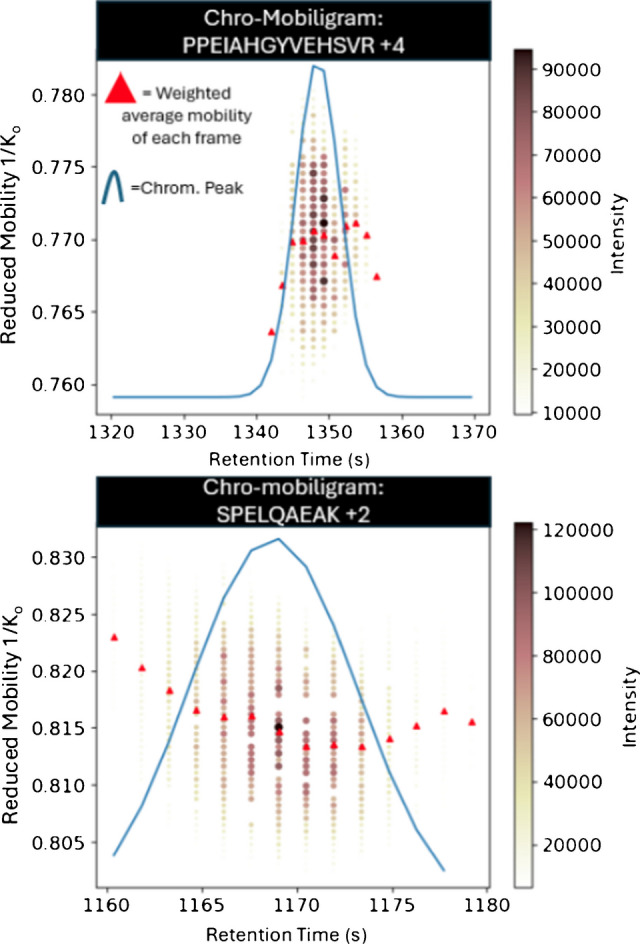
2D chro-mobiligram showing the MS1 measurements at each frame along the elution profile. The *x*-axis represents the elution time in seconds, and the *y*-axis represents the reduced mobility values (1/*K*_0_), while the color of each point is the associated intensity. Overlayed in blue is the corresponding chromatographic peak for reference. Red triangles are the average mobility measurements of each frame, weighted based on intensity. A 3D version, displaying compiled replicates and the full isotopologue envelope, can be found in SI Fig. 2

Given the initial observations of discrepancies, the ion mobility measurements were investigated in greater detail. When examining the data on a per-frame basis, it was occasionally observed that frames near the chromatographic apex did not align with those toward the tail ends. Figure 4 shows two such examples, demonstrating either an ascending or descending shift in average mobility values across IM frames. A possible explanation involves poor ion statistics, which can impact resolution and peak detection, ultimately affecting the resulting mobility measurements.

**Fig. 5:**
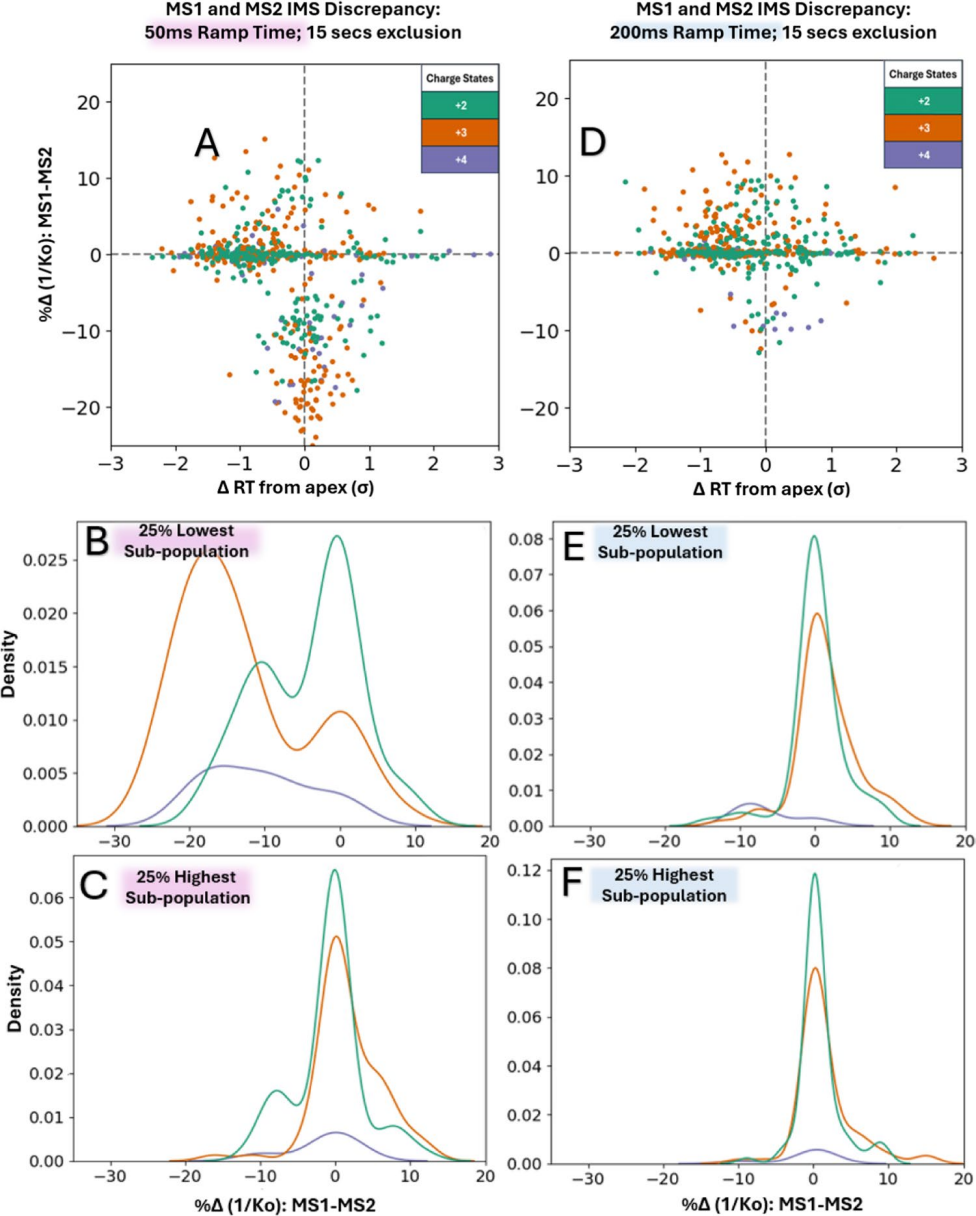
**A**–**F** Comprehensive visualization of discrepancy between MS1- and MS2-associated IMS values during DDA PASEF acquisition in context of chromatographic elution. Each data point represents a MS2 scan resulting in a peptide ID, and its color represents the charge state of the corresponding precursor. The *x*-axis is the time difference between the time of the MS2 scan and the apex of the corresponding chromatographic peak, which will be called (apex ∆T). Delta time is represented as the standard deviation (sigma) of a smoothed Gaussian chromatographic peak to normalize different peak widths. The *y*-axis shows the percent difference between the measured mobility of the MS2 PASEF scans and the consensus MS1 mobility (from IsoPique analysis). Two ramp time settings are compared in the two columns (50 ms and 200 ms). **C**–**F** Visualization of various distributions via density plots of the subpopulations of the 25% highest and lowest intense precursors. The plots for 100 ms ramp time are in SI Fig. 3A

To further investigate alignment of MS1 and MS2 ion mobility measurements on a larger scale, the ion mobility discrepancy (% Δ of MS1-MS2 associated mobilities) for each peptide was plotted relative to the apex of its chromatographic peak (Fig. 5). Increasing the ramp time from 50 to 200 ms resulted in a noticeable decrease in ion mobility (IM) discrepancies, displayed by the different distribution profiles in the density plots (Fig. 5A and D). To assess the impact of precursor intensity, the data were divided into quartiles based on the reported precursor intensity of the corresponding MS2 scan event. A significant number of features, particularly those with + 3 and + 4 charge states, showed negative ∆ % mobility (Fig. 5B and E). In contrast, the top 25% most intense precursors did not display significant deviations in IM discrepancy, demonstrated by the similar distribution profiles in the density plots (Fig. 5C and F). Additionally, the distribution of accumulation times was confirmed to differ between the ramp time settings (SI Fig. 4).

In DDA PASEF, a chromatographic exclusion window (15 s in this study) is applied to promote the sampling of lower-abundance features. However, because IM measurements associated with both precursor and product ions during MS2 events are derived from only a few frames, they lack sufficient sampling across the full elution profile. As a result, these MS2-based IM measurements may not consistently align with the more robust MS1 IM values, which are calculated from multiple frames aggregated over the entire chromatographic peak. To compensate for this limitation, the ramp time is increased, which, for the fluoro-phosphazene tune mix, has been previously reported to linearly improve resolution [47]. Additionally, given a 100% duty cycle, the longer ramp time also increases the accumulation time, resulting in a larger ion packet. Together, these factors facilitate more accurate assignment of MS2-associated ion mobility data that better matches the MS1 ion mobility measurements.

The plus four (+ 4) charge states again stand out as outliers when comparing discrepancies, tending toward negative ∆% mobility and, on average, showing the greatest disagreement with MS1 values. This could be attributed to their complex mobility behavior, which has two effects: (1) it broadens the mobility distribution, reducing the resolution of other species within the mobility space, and (2) it dilutes the signal compared to product ions that exhibit a simpler, single Gaussian mobility profile. Additionally, many points at the extremes of the *y*-axis represent noise captured by the DDA logic. Upon further evaluation, these were identified as artifacts that produce MS2 spectra similar to the peptide but were assigned different MS2-associated ion mobility values.

The effects of different exclusion window durations (0 s, 5 s, and 15 s) were evaluated. When no exclusion window was applied (SI Fig. 3B–C), sampling occurred closer to the peak apex, reducing the typical bias toward the left of the peak apex. However, while this improves sampling precision, it may not be optimal for DDA analysis, as lower-intensity features are more likely to be missed without sufficient exclusion time.

## Future work/conclusions

Beyond improving the accuracy of mobility measurements, several additional factors are critical for effective library building and searching. One consideration is the reproducibility of mobility measurements across different sources and experimental conditions. While repeat sample analyses have been evaluated using IsoPique, inter-day performance under varying calibration conditions is yet to be assessed. Another important aspect is the direct comparison of mobility values obtained from alternative acquisition modes such as PRM and DIA. Ideally, library searching against a DDA-made library should be agnostic of the mode of data acquisition (DDA, PRM, or DIA). This effort contributes to the broader goal of harmonizing analytical methods within the same instrumentation setup. Finally, expanding the library to include more complex analytes (e.g., glycopeptides, which exhibit multiple gas-phase conformers) will further enhance the robustness and applicability of the approach.

This work provides insight into several factors that can influence the experimental accuracy of TIMS mobility values. In the context of library building, typically acquired in DDA mode, it is essential that the inclusion of mobility values be as accurate as possible. It is important to encourage users to adopt precautionary measures—such as proper calibration strategies and careful consideration of instrumental parameters—when utilizing and assigning ion mobility values. The optimized IM settings were tested on a complex biological matrix: tryptic peptides from cNISTmAb. cNISTmAb is a *non-originator* version of the widely used monoclonal antibody (mAb), NISTmAb [48–51] reference material (RM 8671), and is currently under development as a future RM. Overall, this work represents an important step toward incorporating ion mobility metrics into peptide library building, significantly improving the quality of the library and its search results.

## Supplementary Information

Below is the link to the electronic supplementary material.

ESM 1 Supplementary Material 1 (DOCX 1.69 MB)

## Data Availability

ftp://massive-ftp.ucsd.edu/v11/MSV000099983/. Please contact the author(s) for additional data availability.

## Declarations

### Disclaimer

Certain commercial equipment, instruments, or materials are identified in this paper to foster understanding. Such identification does not imply recommendation or endorsement by the National Institute of Standards and Technology, nor does it imply that the materials or equipment identified are necessarily the best available for the purpose.
