# Supplementary material for: Evaluation of TIMS-derived peptide mobility measurements: calibration strategy and MS1/MS2 discrepancies
Source: Anal Bioanal Chem. 2026 Jun 26;418(17):5599–606. doi: 10.1007/s00216-026-06633-9 (PMC13473228; doi:10.1007/s00216-026-06633-9)
Supplement: Supplementary file 1 [file 216_2026_6633_MOESM1_ESM.docx]

Supporting Information

Evaluation of TIMS-Derived Peptide Mobility Measurements: Calibration Strategy and MS1/MS2 Discrepancies

Anh Tran, Tytus Mak, Meghan Burke

Mass Spectrometry Data Center, Biomolecular Measurement Division, National Institute of Standards and Technology (NIST), Gaithersburg, Maryland 20899, United States

Table of contents:

SI Figure 1: An example of two coeluting peptides with overlapping isotopic envelopes that can be resolved with IMS

SI Figure 2: Example of a Chro-mobilgram (Chrono-mobiligram) displaying the MS1 mobility of the isotopologue envelope in 3D space.

SI Figure 3: RT and IMS discrepancies with additional ramp times

SI Figure 4: The reported accumulation time distribution with varying ramp time settings

SI Figure 5: Micro-flow LC gradient details for both TIMS TOF and Agilent 6560 QTOF acquisition

SI Figure 6: Instrument parameters for TIMS-TOF acquisition

SI Figure 7: Instrument parameters for DT-IM QTOF acquisition, with AJS source, and calibration parameters.

SI Table 1: Reference CCS and mobility values used for calibration calculations (positive mode).

SI Table 2: DT-IMS measurements of synthetic peptides in RM8321

SI Figure 1: An example of two coeluting peptides with overlapping isotopic envelopes that can be resolved with IMS. On the right and left are the chromatograms of the two peptides and their isotopologues of the respective feature that were extracted from the middle mobiligram (boxed with dashed texture).

SI Figure 2: Chro-mobilgram (Chrono-mobiligram) displaying the MS1 mobility of the isotopologue envelope centered around the in 3D space. In addition to the retention time and mobility values displayed in the 2D chro-mobiligram, the isotopologue of compiled replicates are shown in the third dimension (z axis: *m/z*). The chro-mobiligram can be used to visualize the tendencies of the mobility to slightly change its distribution over the elution profile.

SI Figure 3: RT and IMS discrepancies with additional ramp times. SI Figure 3A is 100ms ramp time and 15 seconds exclusion window. Discrepancy analysis with 0 exclusion window is also included for reference (SI Figure 3B and SI Figure 3C). The analysis of these figures is explained in the main text.

SI Figure 4: The reported accumulation time distribution with varying ramp time settings. This was extracted from the analysis.tdf of each data file.

SI Figure 5: Micro-flow LC gradient details for both TIMS TOF and Agilent 6560 QTOF acquisition. 15 minutes of equilibrium was used in these runs but is not necessary.

A

B

C

SI Figure 6: Instrument parameters for TIMS-TOF acquisition: (A) Source conditions (B) PASEF acquisition parameters and logic (C) IMS ramps. Crossed out parameters were those irrelevant to the VIP HESI source.

SI Figure 7: Instrument parameters for DT-IM QTOF acquisition, with AJS source, and calibration parameters. During single field calibration, multiple calibrations were performed throughout the run. The following calibration parameters were applied:

For first 3 replicates: Beta: 0.167203; TFix: 0.383906; SE: 0.040177

For last 4 replicates: Beta: 0.168575; TFix: 0.359442; SE: 0.040391

Reference CCS values can be found in SI Table 1.

Additionally, Other experimental parameters include drift temperature range of 270.0 - 272.5 C, drift pressure range of 3.92-3.96 torr, and drift tube length of 78 cm.

SI Table 1: Reference CCS and mobility values used for calibration calculations (positive mode)

|  | m/z | Reference CCS | Reference 1/K (for TIMS) |  |  |
| --- | --- | --- | --- | --- | --- |
|  | 322 | 153.73 | 0.736 |  |  |
|  | 622 | 202.96 | 0.991 |  |  |
|  | 922 | 243.64 | 1.199 |  |  |
|  | 1222 | 282.64 | 1.393 |  |  |
|  | 1522 | 316.96 | 1.568 |  |  |
|  |  |  |  |  |  |

SI Table 2: DT-IMS measurements of synthetic peptides in RM8321 and their associated error

| Z | peptide | mean | std | n | RSD |
| --- | --- | --- | --- | --- | --- |
| 2 | AATVGSLAGQPLQER | 409.3571 | 1.369741 | 7 | 0.334608 |
| 2 | ADLFYDVEALDLESPK | 455.8 | 1.534927 | 6 | 0.336754 |
| 2 | AEDHFSVIDFNQNIR | 442.76 | 1.898157 | 5 | 0.42871 |
| 2 | AEFAEVSK | 316.5857 | 1.274661 | 7 | 0.402628 |
| 2 | AFQPFFVELTMPYSVIR | 493.44 | 1.589969 | 5 | 0.322221 |
| 2 | AGALNSNDAFVLK | 387.8429 | 1.383061 | 7 | 0.356603 |
| 2 | AGDFLEANYMNLQR | 427.1429 | 1.070603 | 7 | 0.250643 |
| 2 | AGKEPGLQIWR | 377.5 | 2.082066 | 5 | 0.551541 |
| 2 | AHVDALR | 310.9 | 1.193315 | 6 | 0.383826 |
| 2 | AIEDYINEFSVR | 402.4857 | 2.102719 | 7 | 0.522433 |
| 2 | AKPALEDLR | 350.95 | 1.55274 | 6 | 0.442439 |
| 2 | ALDLINKR | 342.3143 | 1.392155 | 7 | 0.406689 |
| 2 | ALFVSEEEKK | 381.6667 | 1.42361 | 6 | 0.372998 |
| 2 | ALLVGEHLNIIVTPK | 465.8714 | 1.62554 | 7 | 0.348925 |
| 2 | ALTDMPQMR | 346.4429 | 1.058076 | 7 | 0.305411 |
| 2 | ALVEGVDQLFTDYQIK | 456.5143 | 1.608756 | 7 | 0.3524 |
| 2 | ALVQQMEQLR | 369.6714 | 0.967323 | 7 | 0.261671 |
| 2 | ALYLQYTDETFR | 411.8714 | 1.533747 | 7 | 0.372385 |
| 2 | AMAVEDIISR | 356.7571 | 1.4211 | 7 | 0.398338 |
| 2 | APNHAVVTR | 339.2857 | 1.176759 | 7 | 0.346834 |
| 2 | AQRQVVAGLNFR | 395.1143 | 1.629344 | 7 | 0.412373 |
| 2 | ASEAEDASLLSFMQGYMK | 470.2333 | 1.728198 | 6 | 0.367519 |
| 2 | ASSIIDELFQDR | 389.0857 | 1.818817 | 7 | 0.467459 |
| 2 | ASSIIDELFQDRFFTR | 478.0571 | 2.173214 | 7 | 0.454593 |
| 2 | ASTPNGYDNGIIWATWK | 455.2857 | 1.729575 | 7 | 0.379888 |
| 2 | ATEHLSTLSEK | 377.3 | 1.421267 | 7 | 0.376694 |
| 2 | ATFQTPDFIVPLTDLR | 467.5429 | 1.730951 | 7 | 0.370223 |
| 2 | ATGVLYDYVNK | 376.6286 | 1.22163 | 7 | 0.324359 |
| 2 | ATVVYQGER | 355.4167 | 1.452469 | 6 | 0.408667 |
| 2 | AVMDDFAAFVEK | 389.7333 | 1.196105 | 6 | 0.306903 |
| 2 | AVSMPSFSILGSDVR | 429.0857 | 1.386156 | 7 | 0.323049 |
| 2 | AYKSELEEQLTPVAEETR | 476.6714 | 1.938826 | 7 | 0.406743 |
| 2 | DALSSVQESQVAQQAR | 433.52 | 2.171866 | 5 | 0.500984 |
| 2 | DDNPNLPR | 331.4429 | 1.454713 | 7 | 0.438903 |
| 2 | DFHINLFQVLPWLK | 451.2714 | 2.601099 | 7 | 0.576393 |
| 2 | DGAGDVAFVK | 338.3714 | 1.244607 | 7 | 0.367823 |
| 2 | DGYLFQLLR | 350.6143 | 1.405262 | 7 | 0.4008 |
| 2 | DIFTGLIGPMK | 371.3571 | 1.785924 | 7 | 0.480918 |
| 2 | DINYVNPVIK | 364.5 | 0.898146 | 7 | 0.246405 |
| 2 | DISEVVTPR | 347.4 | 0.737111 | 7 | 0.212179 |
| 2 | DKVNSFFSTFK | 389.4571 | 1.307488 | 7 | 0.335721 |
| 2 | DLATVYVDVLK | 388.36 | 3.021258 | 5 | 0.777953 |
| 2 | DLKVEDIPLAR | 383.9714 | 1.437259 | 7 | 0.374314 |
| 2 | DLMEKVKSPELQAEAK | 460.0143 | 1.895986 | 7 | 0.412158 |
| 2 | DMYSFLEDMGLK | 407.0143 | 1.808709 | 7 | 0.444385 |
| 2 | DNENVVNEYSSELEK | 436.1 | 1.546609 | 6 | 0.354646 |
| 2 | DSAHGFLK | 326.1 | 1.956528 | 6 | 0.599978 |
| 2 | DSGFQMNQLR | 360.48 | 0.980816 | 5 | 0.272086 |
| 2 | DSGRDYVSQFEGSALGK | 451 | 1.839837 | 5 | 0.407946 |
| 2 | DTVIKPLLVEPEGLEK | 468.8429 | 2.21198 | 7 | 0.471796 |
| 2 | DTVQIHDITGK | 369.0714 | 1.554257 | 7 | 0.421126 |
| 2 | DVVLFEK | 316.2167 | 1.268726 | 6 | 0.401221 |
| 2 | DVVLTTTFVDDIK | 431.24 | 1.505988 | 5 | 0.349223 |
| 2 | DWHGVPGQVDAAMAGR | 421.9143 | 1.315838 | 7 | 0.311873 |
| 2 | DYVSQFEGSALGK | 388.7833 | 1.482453 | 6 | 0.381306 |
| 2 | EAQLPVIENK | 362.4857 | 1.065252 | 7 | 0.293874 |
| 2 | EDLIWELLNQAQEHFGK | 490.28 | 2.043771 | 5 | 0.416858 |
| 2 | EDTPNSVWEPAK | 382.6143 | 1.487087 | 7 | 0.388665 |
| 2 | EELLPAQDIK | 366.2571 | 1.350132 | 7 | 0.36863 |
| 2 | EESPLLIGQQSTVSDVPR | 476.1 | 1.443607 | 6 | 0.303215 |
| 2 | EFQLFSSPHGK | 380.3 | 1.161034 | 6 | 0.305294 |
| 2 | ELDESLQVAER | 380.3833 | 1.48784 | 6 | 0.391142 |
| 2 | ELSSFIDK | 331.1667 | 0.950088 | 6 | 0.286891 |
| 2 | ELSYYSLEDLNNK | 422.6857 | 1.47018 | 7 | 0.347819 |
| 2 | EMSGSPASGIPVK | 375.9429 | 2.380376 | 7 | 0.633175 |
| 2 | ENADSLQASLRPHADELK | 471.4857 | 1.50602 | 7 | 0.31942 |
| 2 | EPAHLMSLFGGKPMIIYK | 512.2143 | 1.953995 | 7 | 0.38148 |
| 2 | EPTMYVGSTSVQTSR | 432.4714 | 1.860747 | 7 | 0.430259 |
| 2 | EQLGPVTQEFWDNLEK | 467.4 | 1.886796 | 7 | 0.403679 |
| 2 | ERGHMLENHVER | 402.7143 | 1.292837 | 7 | 0.321031 |
| 2 | ESDTSYVSLK | 357.65 | 1.478851 | 6 | 0.413491 |
| 2 | ESYSGVTLDPR | 380.86 | 1.566206 | 5 | 0.411229 |
| 2 | EVAFDLEIPK | 365.75 | 1.395349 | 6 | 0.381504 |
| 2 | EVDLKDYEDQQK | 405.9333 | 1.438981 | 6 | 0.354487 |
| 2 | EWFWDLATGTMK | 408.6143 | 2.384973 | 7 | 0.583673 |
| 2 | EYVLPSFEVIVEPTEK | 463.6 | 1.656301 | 7 | 0.357269 |
| 2 | FAFNLYR | 331.2286 | 1.177164 | 7 | 0.355393 |
| 2 | FEDGVLDPDYPR | 399.4714 | 1.241926 | 7 | 0.310892 |
| 2 | FLATTPNSLLVSWQPPR | 476.3857 | 2.019429 | 7 | 0.423906 |
| 2 | FMETVAEK | 332.8833 | 1.349691 | 6 | 0.405455 |
| 2 | FNAVLTNPQGDYDTSTGK | 462.2714 | 1.641863 | 7 | 0.355173 |
| 2 | FNKPFVFLMIEQNTK | 462.0286 | 2.134412 | 7 | 0.461965 |
| 2 | FPEVDVLTK | 343.7857 | 1.173923 | 7 | 0.341469 |
| 2 | FPSGTFEQVSQLVK | 425.7429 | 1.520808 | 7 | 0.357213 |
| 2 | FPVEMTHNHNFR | 420.7 | 1.484363 | 7 | 0.352832 |
| 2 | FQNSAILTIQPK | 395.1714 | 1.236547 | 7 | 0.312914 |
| 2 | FSVPAGIVIPSFQALTAR | 464.7429 | 2.526432 | 7 | 0.543619 |
| 2 | FSVVYAK | 313.0143 | 1.375292 | 7 | 0.43937 |
| 2 | FSYSKNETYQLFLSYSSK | 506.58 | 2.552842 | 5 | 0.503937 |
| 2 | FSYSSGHVHLSSENK | 442.72 | 1.507315 | 5 | 0.340467 |
| 2 | FTNIGPDTMR | 358 | 1.461164 | 5 | 0.408146 |
| 2 | FTVDRPFLFLIYEHR | 480.4571 | 1.861771 | 7 | 0.3875 |
| 2 | FVTWIEGVMR | 366.4857 | 1.514847 | 7 | 0.413344 |
| 2 | FYNQVSTPLLR | 383.5429 | 1.214986 | 7 | 0.31678 |
| 2 | GAYPLSIEPIGVR | 393.7571 | 1.048582 | 7 | 0.266302 |
| 2 | GDKVWVYPPEK | 386.9833 | 1.437243 | 6 | 0.371397 |
| 2 | GDSGGPLIVHKR | 378.0429 | 5.933761 | 7 | 1.5696 |
| 2 | GETHEQVHSILHFK | 450.4167 | 1.616684 | 6 | 0.358931 |
| 2 | GEVQAMLGQSTEELR | 425.7143 | 1.295413 | 7 | 0.304292 |
| 2 | GEVQAMLGQSTEELRVR | 506 | 1.704895 | 7 | 0.336936 |
| 2 | GEWVALNPLR | 363.0286 | 1.965294 | 7 | 0.541361 |
| 2 | GFEPTLEALFGK | 380.3571 | 1.58835 | 7 | 0.417594 |
| 2 | GFSLDEATNLNGGLLR | 439.1714 | 2.127373 | 7 | 0.484406 |
| 2 | GGEGTGYFVDFSVR | 399.4 | 1.571623 | 7 | 0.393496 |
| 2 | GHLFLQTDQPIYNPGQR | 478.9333 | 1.194432 | 6 | 0.249394 |
| 2 | GSFEFPVGDAVSK | 391.4571 | 1.306212 | 7 | 0.33368 |
| 2 | GSPAINVAVHVFR | 423.1714 | 1.059425 | 7 | 0.250354 |
| 2 | GSPAINVAVHVFRK | 435.9143 | 1.749694 | 7 | 0.401385 |
| 2 | GWVTDGFSSLK | 370 | 0.834666 | 7 | 0.225585 |
| 2 | GWVTDGFSSLKDYWSTVK | 488.7857 | 2.181306 | 7 | 0.44627 |
| 2 | GYSIFSYATK | 355.4857 | 1.096097 | 7 | 0.308338 |
| 2 | HGGLYHENMR | 371.8857 | 1.103674 | 7 | 0.296778 |
| 2 | HGTDDGVVWMNWK | 411.2286 | 1.290626 | 7 | 0.313846 |
| 2 | HLEVDVWVIEPQGLR | 451.2429 | 1.522998 | 7 | 0.337512 |
| 2 | HLSLLTTLSNR | 386.6286 | 1.253946 | 7 | 0.324328 |
| 2 | HLVPGAPFLLQALVR | 442 | 1.322876 | 7 | 0.299293 |
| 2 | HPDYSVVLLLR | 392.6286 | 1.518458 | 7 | 0.386742 |
| 2 | HQLYIDETVNSNIPTNLR | 486.2714 | 1.878576 | 7 | 0.386323 |
| 2 | HSTIFENLANK | 373.4571 | 1.499841 | 7 | 0.40161 |
| 2 | HTLNQIDEVK | 379.3571 | 1.569349 | 7 | 0.413686 |
| 2 | HVVPNEVVVQR | 382.82 | 1.783816 | 5 | 0.465967 |
| 2 | HYQINQQWER | 393.2667 | 2.307091 | 6 | 0.586648 |
| 2 | IADAHLDR | 333.2167 | 1.663029 | 6 | 0.499083 |
| 2 | IAFSATR | 309.9714 | 1.390101 | 7 | 0.448461 |
| 2 | IAQWQSFQLEGGLK | 428.1857 | 1.604606 | 7 | 0.374745 |
| 2 | IEGNLIFDPNNYLPK | 447.5 | 1.552417 | 7 | 0.346909 |
| 2 | IHWESASLLR | 365.2429 | 1.155937 | 7 | 0.316485 |
| 2 | IIVPLNNR | 341.3571 | 1.035788 | 7 | 0.303432 |
| 2 | IKVLNQELR | 370 | 1.378405 | 7 | 0.372542 |
| 2 | IPIEDGSGEVVLSR | 405.4143 | 1.252236 | 7 | 0.308878 |
| 2 | IPLDLVPK | 336.5857 | 1.13934 | 7 | 0.338499 |
| 2 | IQPSGGTNINEALLR | 424.9286 | 1.299634 | 7 | 0.305848 |
| 2 | ISASAEELR | 335.7857 | 1.589999 | 7 | 0.473516 |
| 2 | ISEGLPALEFPNEK | 412.8 | 1.574802 | 7 | 0.381493 |
| 2 | ITENDIQIALDDAK | 415.7571 | 1.101298 | 7 | 0.26489 |
| 2 | ITPNLAEFAFSLYR | 429.4714 | 2.335747 | 7 | 0.543866 |
| 2 | IVSSAMEPDR | 345.1429 | 1.332738 | 7 | 0.386141 |
| 2 | IYGNQDTSSQLKK | 415.0333 | 1.120119 | 6 | 0.269887 |
| 2 | IYHSHIDAPK | 377.4286 | 1.101082 | 7 | 0.291732 |
| 2 | IYISGMAPRPSLAK | 448.2714 | 1.451108 | 7 | 0.323712 |
| 2 | KAMAVEDIISR | 372.6 | 1.36626 | 7 | 0.366683 |
| 2 | KDNEQHVFK | 354.9571 | 0.793425 | 7 | 0.223527 |
| 2 | KFPSGTFEQVSQLVK | 445.5143 | 1.309489 | 7 | 0.293928 |
| 2 | KGEWVALNPLR | 379.6429 | 0.95369 | 7 | 0.251207 |
| 2 | KGEWVALNPLRK | 411.3143 | 1.325932 | 7 | 0.322365 |
| 2 | KLSSWVLLMK | 376.7571 | 1.587301 | 7 | 0.421306 |
| 2 | KLVPFATELHER | 408.6286 | 1.309853 | 7 | 0.320549 |
| 2 | KLWAYLTINQLLAER | 466.4667 | 1.610797 | 6 | 0.345319 |
| 2 | KSASDLTWDNLK | 396.7429 | 1.494275 | 7 | 0.376636 |
| 2 | KTLLSNLEEAK | 391.7857 | 1.350837 | 7 | 0.34479 |
| 2 | KTLLSNLEEAKK | 400.7857 | 1.39932 | 7 | 0.349144 |
| 2 | KVEQAVETEPEPELR | 440.8 | 1.555635 | 5 | 0.352912 |
| 2 | KVPQVSTPTLVEVSR | 436.7 | 1.454877 | 7 | 0.333152 |
| 2 | KWQEEMELYR | 402.1143 | 1.173923 | 7 | 0.291938 |
| 2 | KYFIDFVAR | 368.6571 | 1.093705 | 7 | 0.296673 |
| 2 | KYNSQNQSNNQFVLYR | 469.9143 | 2.502951 | 7 | 0.53264 |
| 2 | LANLTQGEDQYYLR | 447.5714 | 0.939351 | 7 | 0.209877 |
| 2 | LAVYQAGAR | 324.4286 | 1.209289 | 7 | 0.372744 |
| 2 | LDAQASFLPK | 352.9143 | 1.496026 | 7 | 0.423906 |
| 2 | LDEVKEQVAEVR | 402.18 | 1.505656 | 5 | 0.374374 |
| 2 | LDGKFSVVYAK | 385.4857 | 1.535609 | 7 | 0.398357 |
| 2 | LFDSDPITVTVPVEVSRK | 477.6571 | 1.258117 | 7 | 0.263393 |
| 2 | LGPHAGDVEGHLSFLEK | 461.1286 | 1.946548 | 7 | 0.422127 |
| 2 | LGPLVEQGR | 329.6833 | 1.371739 | 6 | 0.416078 |
| 2 | LGQYASPTAK | 346.5 | 0.613188 | 6 | 0.176966 |
| 2 | LHEAFSPVSYQHDLALLR | 496.7833 | 1.723272 | 6 | 0.346886 |
| 2 | LHIMAGR | 313.5 | 1.371131 | 7 | 0.437362 |
| 2 | LKEEIGKELEELR | 445.9143 | 1.383749 | 7 | 0.310317 |
| 2 | LKNSLFEYQK | 385.7571 | 1.876928 | 7 | 0.486557 |
| 2 | LKSWFEPLVEDMQR | 449.6 | 1.688194 | 7 | 0.375488 |
| 2 | LLDNWDSVTSTFSK | 427.9286 | 2.254414 | 7 | 0.52682 |
| 2 | LLIYAVLPTGDVIGDSAK | 475.7 | 2.759227 | 7 | 0.580035 |
| 2 | LNAENNATFYFK | 398.1 | 1.322876 | 7 | 0.332297 |
| 2 | LPPNVVEESAR | 361.3 | 1.188837 | 7 | 0.329044 |
| 2 | LQGTLPVEAR | 362.0857 | 1.737267 | 7 | 0.479794 |
| 2 | LQHLENELTHDIITK | 451.3429 | 1.623635 | 7 | 0.359734 |
| 2 | LREQLGPVTQEFWDNLEK | 500.1833 | 1.615446 | 6 | 0.322971 |
| 2 | LRTEGDGVYTLNDKK | 437.06 | 1.357571 | 5 | 0.310614 |
| 2 | LSINTHPSQKPLSITVR | 468.9286 | 2.026256 | 7 | 0.432103 |
| 2 | LSNENHGIAQR | 375.9429 | 1.298534 | 7 | 0.345407 |
| 2 | LSPIYNLVPVK | 375.8429 | 1.39983 | 7 | 0.372451 |
| 2 | LSPLGEEMR | 341.22 | 1.380942 | 5 | 0.404707 |
| 2 | LSSPAVITDK | 344.0833 | 1.427469 | 6 | 0.414861 |
| 2 | LTIGEGQQHHLGGAK | 433.2 | 1.741264 | 6 | 0.401954 |
| 2 | LVAYYTLIGASGQR | 416.5 | 1.388044 | 7 | 0.333264 |
| 2 | LVNEVTEFAK | 359.4 | 1.027943 | 7 | 0.286016 |
| 2 | LVTDLTK | 307.9143 | 1.234812 | 7 | 0.401025 |
| 2 | LWAYLTIQELLAK | 449.7667 | 1.544884 | 6 | 0.343486 |
| 2 | MATTMIQSK | 339.1429 | 1.49873 | 7 | 0.441917 |
| 2 | MGPTELLIEMEDWK | 439.76 | 1.297305 | 5 | 0.295003 |
| 2 | MGPTELLIEMEDWKGDK | 489.2571 | 2.294092 | 7 | 0.468893 |
| 2 | MKGLIDEVNQDFTNR | 451.8714 | 1.515161 | 7 | 0.335308 |
| 2 | MKPVPDLVPGNFK | 406.7571 | 0.842332 | 7 | 0.207085 |
| 2 | MLTPEHVFIHPGWK | 454.6857 | 1.519242 | 7 | 0.33413 |
| 2 | MYLGYEYVTAIR | 406.5167 | 1.616684 | 6 | 0.397692 |
| 2 | NFPSPVDAAFR | 363.8143 | 1.264158 | 7 | 0.347473 |
| 2 | NKPGVYTDVAYYLAWIR | 471.8 | 1.392839 | 7 | 0.295218 |
| 2 | NNKDSHSLTTNIMEILR | 476.4286 | 1.432613 | 7 | 0.300698 |
| 2 | NPANPVQR | 325.4571 | 1.312577 | 7 | 0.403302 |
| 2 | NPNLPPETVDSLK | 397.9429 | 1.058076 | 7 | 0.265886 |
| 2 | NSLFEYQK | 346.56 | 1.613382 | 5 | 0.465542 |
| 2 | NWGLSVYADKPETTK | 461.7833 | 1.907267 | 6 | 0.413022 |
| 2 | NWIQYK | 320.18 | 1.782414 | 5 | 0.556691 |
| 2 | NYNLVESLK | 363 | 1.075484 | 7 | 0.296277 |
| 2 | PLVEEPQNLIK | 395.9429 | 1.198412 | 7 | 0.302673 |
| 2 | PNSMVVEHPEFLK | 428.55 | 1.30499 | 6 | 0.304513 |
| 2 | RHPDYSVVLLLR | 417.5429 | 1.459289 | 7 | 0.349494 |
| 2 | RHPYFYAPELLFFAK | 473.1143 | 4.662413 | 7 | 0.985473 |
| 2 | RLEVDIDIK | 353.6857 | 0.99403 | 7 | 0.281049 |
| 2 | RPYFPVAVGK | 364.9714 | 1.245946 | 7 | 0.341382 |
| 2 | RQDNEILIFWSK | 418.7714 | 1.759464 | 7 | 0.420149 |
| 2 | RQSEDSTFYLGER | 411.9714 | 1.679994 | 7 | 0.407794 |
| 2 | RRDGYLFQLLR | 409.8286 | 1.117608 | 7 | 0.272701 |
| 2 | RSFFSFLGEAFDGAR | 439.2857 | 1.33095 | 7 | 0.302981 |
| 2 | RTHLPEVFLSK | 391.5429 | 1.577822 | 7 | 0.402976 |
| 2 | RTHLPEVFLSKVLEPTLK | 506.84 | 1.807761 | 5 | 0.356673 |
| 2 | RVDTVDPPYPR | 386.8 | 1.007472 | 5 | 0.260463 |
| 2 | RVEPYGENFNK | 385.7833 | 1.213947 | 6 | 0.314671 |
| 2 | RYIETDPANR | 369.2 | 0.774597 | 7 | 0.209804 |
| 2 | SDVVYTDWK | 355.58 | 1.047378 | 5 | 0.294555 |
| 2 | SELEEQLTPVAEETR | 438.4 | 1.357694 | 7 | 0.309693 |
| 2 | SELTQQLNALFQDK | 429.8857 | 1.531262 | 7 | 0.356202 |
| 2 | SFFSFLGEAFDGAR | 417.8 | 1.50333 | 7 | 0.35982 |
| 2 | SGFPQVSMFFTHTFPK | 492.4857 | 1.591046 | 7 | 0.323064 |
| 2 | SGVQQLIQYYQDQK | 438.0714 | 1.718249 | 7 | 0.39223 |
| 2 | SHALQLNNR | 345.4 | 1.066146 | 7 | 0.30867 |
| 2 | SIEVFGQFNGK | 368.4714 | 0.996183 | 7 | 0.270356 |
| 2 | SIEVFGQFNGKR | 407.4833 | 1.340771 | 6 | 0.329037 |
| 2 | SKEQLTPLIKK | 387.84 | 1.291511 | 5 | 0.333001 |
| 2 | SLAELGGHLDQQVEEFRR | 487.3667 | 1.94799 | 6 | 0.399697 |
| 2 | SLHTLFGDK | 346.1 | 1.341641 | 6 | 0.387645 |
| 2 | SPELQAEAK | 328.3571 | 1.157378 | 7 | 0.352475 |
| 2 | SPELQAEAKSYFEK | 445.1857 | 1.493797 | 7 | 0.335545 |
| 2 | SSALDMENFR | 364.7333 | 1.466515 | 6 | 0.402079 |
| 2 | SSNLIILEEHLK | 416.8429 | 7.51706 | 7 | 1.803332 |
| 2 | SVLGQLGITK | 345.0571 | 0.95369 | 7 | 0.276386 |
| 2 | SVVDENFSWYLEDNIK | 459.9857 | 1.679711 | 7 | 0.365166 |
| 2 | SWFEPLVEDMQR | 409.1167 | 1.747474 | 6 | 0.427134 |
| 2 | SYFEKSKEQLTPLIK | 467.8286 | 1.816328 | 7 | 0.388247 |
| 2 | SYFPESWLWEVHLVPR | 487.5167 | 2.159089 | 6 | 0.442875 |
| 2 | SYTITGLQPGTDYK | 412.7 | 1.428286 | 7 | 0.346083 |
| 2 | TAAQNLYEKTYLPAVDEK | 484.9429 | 1.794303 | 7 | 0.370003 |
| 2 | TAGWNIPMGLLYNK | 421.2 | 1.75784 | 7 | 0.417341 |
| 2 | TDAPDLPEENQAR | 391.3333 | 1.686021 | 6 | 0.43084 |
| 2 | TEDTIFLR | 338.1429 | 1.43278 | 7 | 0.42372 |
| 2 | TEGDGVYTLNDK | 381.8333 | 1.924231 | 6 | 0.503945 |
| 2 | TEHPFTVEEFVLPK | 433.9714 | 1.0291 | 7 | 0.237136 |
| 2 | TEHYEEQIEAFK | 414.0571 | 1.740553 | 7 | 0.420365 |
| 2 | TELRPGETLNVNFLLR | 464.9714 | 1.352423 | 7 | 0.290861 |
| 2 | TEVNVLPGAK | 337.7 | 1.770593 | 5 | 0.524309 |
| 2 | TGAQELLR | 326.9143 | 1.381338 | 7 | 0.422538 |
| 2 | THLAPYSDELR | 383.18 | 1.849865 | 5 | 0.482767 |
| 2 | THLPEVFLSK | 369.5286 | 1.531572 | 7 | 0.414467 |
| 2 | THLPEVFLSKVLEPTLK | 516 | 2.115813 | 7 | 0.410041 |
| 2 | TLLPVSKPEIR | 402.4857 | 1.213025 | 7 | 0.301383 |
| 2 | TLLSNLEEAK | 375.8286 | 1.574499 | 7 | 0.418941 |
| 2 | TMEQFTIHLTVNPQSK | 485.4571 | 1.333988 | 7 | 0.27479 |
| 2 | TMTIHNGMFFSTYDR | 450.7 | 1.429452 | 7 | 0.317163 |
| 2 | TNFDNDIALVR | 380.1714 | 1.27895 | 7 | 0.336414 |
| 2 | TPSAAYLWVGTGASEAEK | 454.1286 | 1.542416 | 7 | 0.339643 |
| 2 | TSNFNAAISLK | 369.6571 | 0.97785 | 7 | 0.264529 |
| 2 | TTIEKPVWLGFLGPIIK | 478.2833 | 2.229275 | 6 | 0.466099 |
| 2 | TTNIQGINLLFSSR | 417.8286 | 1.760411 | 7 | 0.421324 |
| 2 | TVGSDTFYSFK | 369.9286 | 1.195428 | 7 | 0.323151 |
| 2 | TVIGPDGHK | 324.5714 | 1.260574 | 7 | 0.388381 |
| 2 | TVMVNIENPEGIPVK | 431.3286 | 1.453403 | 7 | 0.33696 |
| 2 | TWRNDLISATK | 382.5714 | 1.65903 | 7 | 0.433652 |
| 2 | TYETTLEK | 337.8286 | 1.682968 | 7 | 0.498172 |
| 2 | TYLPAVDEKLR | 403.8429 | 1.34022 | 7 | 0.331867 |
| 2 | TYLPAVDEKLRDLYSK | 500.0714 | 1.893598 | 7 | 0.378666 |
| 2 | TYMLAFDVNDEK | 407.2714 | 1.268482 | 7 | 0.311459 |
| 2 | TYNVLDMK | 340.7714 | 1.391299 | 7 | 0.408279 |
| 2 | VDKDNEDFQESNR | 417.76 | 1.318332 | 5 | 0.315572 |
| 2 | VDTVDPPYPR | 358.8167 | 2.350674 | 6 | 0.655118 |
| 2 | VDVIPVNLPGEHGQR | 429.5 | 1.478738 | 7 | 0.344293 |
| 2 | VEDPESTLFGSVIR | 415.3857 | 1.748741 | 7 | 0.420992 |
| 2 | VFDEFKPLVEEPQNLIK | 484.4571 | 1.721295 | 7 | 0.355304 |
| 2 | VFSNGADLSGVTEEAPLK | 450.7571 | 1.847392 | 7 | 0.409842 |
| 2 | VGDTLNLNLR | 355.4286 | 1.210667 | 7 | 0.340622 |
| 2 | VGFYESDVMGR | 373.3143 | 1.215769 | 7 | 0.325669 |
| 2 | VGYVSGWGR | 337.2167 | 1.020621 | 6 | 0.30266 |
| 2 | VIGNMGQTMEQLTPELK | 458.4429 | 1.983143 | 7 | 0.432582 |
| 2 | VKDISEVVTPR | 385.5571 | 1.255464 | 7 | 0.325623 |
| 2 | VKDLATVYVDVLK | 418.2143 | 1.491564 | 7 | 0.356651 |
| 2 | VKSPELQAEAKSYFEK | 465.2286 | 1.752821 | 7 | 0.376766 |
| 2 | VLEPTLK | 310.5143 | 1.225328 | 7 | 0.394612 |
| 2 | VLNQELR | 332.0571 | 1.426951 | 7 | 0.429731 |
| 2 | VLSLAQEQVGGSPEK | 416.1857 | 1.392155 | 7 | 0.334503 |
| 2 | VMDKYTFELSR | 398.6857 | 1.062118 | 7 | 0.266405 |
| 2 | VPGLYYFTYHASSR | 446.3286 | 1.313664 | 7 | 0.294327 |
| 2 | VPGTSTSATLTGLTR | 407.7571 | 0.888552 | 7 | 0.217912 |
| 2 | VPLLLSEPINIIDALEMR | 492.6571 | 1.515319 | 7 | 0.307581 |
| 2 | VPQVSTPTLVEVSR | 411.3 | 1.177568 | 7 | 0.286304 |
| 2 | VQFELHYQEVK | 409.4714 | 1.333809 | 7 | 0.325739 |
| 2 | VQPYLDDFQK | 376.7286 | 1.499683 | 7 | 0.39808 |
| 2 | VRGGEGTGYFVDFSVR | 441.0571 | 1.251475 | 7 | 0.283744 |
| 2 | VSFLSALEEYTK | 397.2857 | 1.278578 | 7 | 0.321828 |
| 2 | VTFQLTYEEVLK | 415.32 | 2.320991 | 5 | 0.558844 |
| 2 | VTIMWTPPESAVTGYR | 443.2429 | 1.537159 | 7 | 0.346798 |
| 2 | VTWAPPPSIDLTNFLVR | 470.3857 | 1.792976 | 7 | 0.381171 |
| 2 | VVGGLVALR | 331.0143 | 1.524717 | 7 | 0.46062 |
| 2 | VVLHPNYSQVDIGLIK | 471.8143 | 2.043223 | 7 | 0.433057 |
| 2 | VYSLNDDLKPAK | 402.9571 | 1.83017 | 7 | 0.454185 |
| 2 | WFYIASAFR | 366.5143 | 1.382544 | 7 | 0.377214 |
| 2 | WFYIASAFRNEEYNK | 483.9 | 2.471437 | 6 | 0.510733 |
| 2 | WKNFPSPVDAAFR | 416.9571 | 1.662184 | 7 | 0.398646 |
| 2 | WLPSSSPVTGYR | 380.3714 | 1.265852 | 7 | 0.332794 |
| 2 | WQEEMELYR | 381.3833 | 1.222157 | 6 | 0.320454 |
| 2 | WYEIEKIPTTFENGR | 456.2 | 1.882551 | 6 | 0.412659 |
| 2 | YEASILTHDSSIR | 406.1286 | 1.332559 | 7 | 0.328113 |
| 2 | YEFLNGR | 332.2667 | 1.174166 | 6 | 0.353381 |
| 2 | YEITTIHNLFR | 418.2143 | 1.481473 | 7 | 0.354238 |
| 2 | YFIDFVAR | 355.0714 | 1.597617 | 7 | 0.449943 |
| 2 | YGLVTYATYPK | 387.8714 | 1.239239 | 7 | 0.319497 |
| 2 | YGMVAQVTQTLK | 397.3143 | 1.353303 | 7 | 0.340613 |
| 2 | YKEENDDFASFRVDR | 454.3143 | 1.697477 | 7 | 0.373635 |
| 2 | YLGEEYVK | 345.9143 | 1.429952 | 7 | 0.413383 |
| 2 | YLYEIAR | 337.8 | 1.89473 | 7 | 0.560903 |
| 2 | YQISVNK | 319.2143 | 1.365563 | 7 | 0.427789 |
| 2 | YTFELSR | 334.8429 | 1.142887 | 7 | 0.34132 |
| 2 | YVGGQEHFAHLLILR | 464.7667 | 1.945936 | 6 | 0.418691 |
| 2 | YVLPNFEVK | 355.2286 | 1.299634 | 7 | 0.365858 |
| 2 | YVNKEIQNAVNGVK | 428.0143 | 1.664761 | 7 | 0.38895 |
| 2 | YYTYLIMNK | 374.6714 | 0.892028 | 7 | 0.238083 |
| 3 | AGDFLEANYMNLQR | 488.12 | 3.105962 | 5 | 0.636311 |
| 3 | AGKEPGLQIWR | 451.2 | 1.768238 | 7 | 0.391897 |
| 3 | AKPALEDLR | 441.48 | 2.075331 | 5 | 0.470085 |
| 3 | ALDLINKR | 418.7 | 2.167948 | 5 | 0.517781 |
| 3 | ALFVSEEEKK | 436.18 | 1.613072 | 5 | 0.369818 |
| 3 | ALLVGEHLNIIVTPK | 512.2714 | 1.3865 | 7 | 0.270657 |
| 3 | ALYLQYTDETFR | 480.38 | 1.947306 | 5 | 0.405368 |
| 3 | APNHAVVTR | 434.9 | 1.922672 | 7 | 0.442095 |
| 3 | AQRQVVAGLNFR | 447.4571 | 1.806338 | 7 | 0.40369 |
| 3 | ASEAEDASLLSFMQGYMK | 559.95 | 1.825103 | 6 | 0.32594 |
| 3 | ASSIIDELFQDRFFTR | 512.5571 | 2.172447 | 7 | 0.423845 |
| 3 | ASTPNGYDNGIIWATWK | 531.88 | 2.727086 | 5 | 0.512726 |
| 3 | ATEHLSTLSEK | 429.9 | 1.937782 | 5 | 0.450752 |
| 3 | AVSMPSFSILGSDVR | 507.6 | 3.510556 | 6 | 0.691599 |
| 3 | AYKSELEEQLTPVAEETR | 534.9857 | 1.959106 | 7 | 0.366198 |
| 3 | DFHINLFQVLPWLK | 506.7 | 2.189749 | 5 | 0.432159 |
| 3 | DKVNSFFSTFK | 453.9667 | 2.023528 | 6 | 0.445744 |
| 3 | DLEIEVVLFHPNYNINGK | 571.38 | 1.512283 | 5 | 0.264672 |
| 3 | DLMEKVKSPELQAEAK | 508.1857 | 2.839265 | 7 | 0.558706 |
| 3 | DRLDEVKEQVAEVR | 503.7571 | 1.855494 | 7 | 0.368331 |
| 3 | DSAHGFLK | 420.64 | 1.862257 | 5 | 0.44272 |
| 3 | DSQEEEKTEALTSAKR | 520.3 | 1.871897 | 6 | 0.359773 |
| 3 | DTVIKPLLVEPEGLEK | 544.9143 | 2.29378 | 7 | 0.420943 |
| 3 | DTVQIHDITGK | 435.9143 | 1.708243 | 7 | 0.391876 |
| 3 | DWHGVPGQVDAAMAGR | 476.0286 | 1.439577 | 7 | 0.302414 |
| 3 | EDLIWELLNQAQEHFGK | 551.3286 | 2.581804 | 7 | 0.468288 |
| 3 | EESPLLIGQQSTVSDVPR | 534.46 | 2.124382 | 5 | 0.397482 |
| 3 | EPAHLMSLFGGKPMIIYK | 550.5143 | 1.7535 | 7 | 0.31852 |
| 3 | EQLGPVTQEFWDNLEK | 520.0833 | 2.31898 | 6 | 0.445886 |
| 3 | ERGHMLENHVER | 471.2 | 1.18603 | 7 | 0.251704 |
| 3 | EVDLKDYEDQQK | 503.3167 | 1.892529 | 6 | 0.376012 |
| 3 | FLATTPNSLLVSWQPPR | 559.6 | 2.161789 | 7 | 0.38631 |
| 3 | FQNSAILTIQPK | 482.78 | 1.814387 | 5 | 0.375821 |
| 3 | FSVPAGIVIPSFQALTAR | 583.2857 | 4.879012 | 7 | 0.83647 |
| 3 | FSYSKNETYQLFLSYSSK | 533.6857 | 2.002855 | 7 | 0.375287 |
| 3 | FSYSSGHVHLSSENK | 509.4833 | 1.956953 | 6 | 0.384105 |
| 3 | FTVDRPFLFLIYEHR | 555.2143 | 1.837053 | 7 | 0.330873 |
| 3 | GDKVWVYPPEK | 459.2833 | 2.095153 | 6 | 0.456179 |
| 3 | GDKVWVYPPEKK | 463.46 | 2.41723 | 5 | 0.521562 |
| 3 | GETHEQVHSILHFK | 493.1 | 2.168717 | 7 | 0.439813 |
| 3 | GEVQAMLGQSTEELRVR | 536.0143 | 1.405262 | 7 | 0.262169 |
| 3 | GFEPTLEALFGK | 469.76 | 1.835211 | 5 | 0.39067 |
| 3 | GFSLDEATNLNGGLLR | 517.38 | 2.678992 | 5 | 0.5178 |
| 3 | GHLFLQTDQPIYNPGQR | 519 | 1.881489 | 7 | 0.362522 |
| 3 | GHMLENHVER | 447.84 | 1.734359 | 5 | 0.387272 |
| 3 | GSPAINVAVHVFR | 470.4857 | 1.42177 | 7 | 0.302192 |
| 3 | GSPAINVAVHVFRK | 485.0833 | 2.269288 | 6 | 0.467814 |
| 3 | GSWVNKFPVEMTHNHNFR | 569.36 | 1.921718 | 5 | 0.337522 |
| 3 | GWVTDGFSSLKDYWSTVK | 536.0714 | 6.897515 | 7 | 1.286678 |
| 3 | HGGLYHENMR | 474.6 | 1.516575 | 7 | 0.319548 |
| 3 | HGTDDGVVWMNWK | 502.26 | 1.066771 | 5 | 0.212394 |
| 3 | HLEVDVWVIEPQGLR | 534.2714 | 2.071806 | 7 | 0.387782 |
| 3 | HLVPGAPFLLQALVR | 547.9857 | 1.881868 | 7 | 0.343416 |
| 3 | HQLYIDETVNSNIPTNLR | 587.0571 | 1.992366 | 7 | 0.339382 |
| 3 | HSIFTPETNPR | 468.22 | 2.686447 | 5 | 0.573758 |
| 3 | HTLNQIDEVK | 440.8 | 2.831078 | 5 | 0.642259 |
| 3 | IADAHLDR | 425.3333 | 1.950043 | 6 | 0.458474 |
| 3 | IADNKQSSFK | 433.68 | 1.336787 | 5 | 0.308243 |
| 3 | IEGNLIFDPNNYLPK | 521.68 | 1.768333 | 5 | 0.338969 |
| 3 | IIRSSEDPNEDIVER | 483.7167 | 2.353225 | 6 | 0.486488 |
| 3 | IKVLNQELR | 444.7167 | 1.849775 | 6 | 0.415945 |
| 3 | IPIEDGSGEVVLSR | 494.02 | 1.813009 | 5 | 0.366991 |
| 3 | IQPSGGTNINEALLR | 485.6 | 1.036822 | 5 | 0.213514 |
| 3 | ITENDIQIALDDAK | 487.52 | 1.676902 | 5 | 0.343966 |
| 3 | ITPNLAEFAFSLYR | 522.2167 | 1.698725 | 6 | 0.325291 |
| 3 | IYGNQDTSSQLKK | 473.34 | 2.370232 | 5 | 0.500746 |
| 3 | IYISGMAPRPSLAK | 507.1571 | 1.350132 | 7 | 0.266216 |
| 3 | KGEWVALNPLR | 474.8667 | 0.84538 | 6 | 0.178025 |
| 3 | KGEWVALNPLRK | 477.9 | 1.671327 | 7 | 0.349723 |
| 3 | KLSSWVLLMK | 441.8333 | 2.968277 | 6 | 0.671809 |
| 3 | KLVPFATELHER | 500.0143 | 1.82522 | 7 | 0.365034 |
| 3 | KLWAYLTINQLLAER | 511.6333 | 2.023528 | 6 | 0.395504 |
| 3 | KTLLSNLEEAK | 440.4429 | 1.352599 | 7 | 0.3071 |
| 3 | KTLLSNLEEAKK | 453.2143 | 2.114575 | 7 | 0.466573 |
| 3 | KVPQVSTPTLVEVSR | 477.1571 | 1.691998 | 7 | 0.3546 |
| 3 | KWQEEMELYR | 460.36 | 1.814663 | 5 | 0.394183 |
| 3 | KYFIDFVAR | 440.96 | 1.543697 | 5 | 0.350076 |
| 3 | KYNSQNQSNNQFVLYR | 501.9714 | 2.818223 | 7 | 0.561431 |
| 3 | LDELRDEGK | 423.36 | 2.082787 | 5 | 0.491966 |
| 3 | LDGKFSVVYAK | 466.5714 | 1.731776 | 7 | 0.371171 |
| 3 | LFDSDPITVTVPVEVSRK | 581.2571 | 1.096748 | 7 | 0.188686 |
| 3 | LGPHAGDVEGHLSFLEK | 542.7 | 1.87261 | 7 | 0.345054 |
| 3 | LGVRPSQGGEAPR | 462.28 | 1.2498 | 5 | 0.270356 |
| 3 | LHEAFSPVSYQHDLALLR | 542.8143 | 1.819733 | 7 | 0.33524 |
| 3 | LKEEIGKELEELR | 494.9143 | 1.418248 | 7 | 0.286564 |
| 3 | LKNSLFEYQK | 465.6286 | 2.196751 | 7 | 0.471782 |
| 3 | LKSWFEPLVEDMQR | 493.7143 | 2.329827 | 7 | 0.471898 |
| 3 | LLPHANEVSQK | 447.66 | 1.813284 | 5 | 0.405058 |
| 3 | LQHLENELTHDIITK | 518.9 | 1.17047 | 7 | 0.225568 |
| 3 | LRDLYSK | 416.66 | 2.397499 | 5 | 0.575409 |
| 3 | LREQLGPVTQEFWDNLEK | 566.7714 | 2.369046 | 7 | 0.41799 |
| 3 | LRTEGDGVYTLNDKK | 522.6429 | 2.023905 | 7 | 0.387244 |
| 3 | LSINTHPSQKPLSITVR | 542.6333 | 2.537453 | 6 | 0.467618 |
| 3 | LSNENHGIAQR | 452.2286 | 1.436099 | 7 | 0.31756 |
| 3 | LTIGEGQQHHLGGAK | 479.24 | 3.216053 | 5 | 0.671074 |
| 3 | LVAYYTLIGASGQR | 502.2571 | 1.633868 | 7 | 0.325305 |
| 3 | LVDKFLEDVKK | 448.7833 | 1.53547 | 6 | 0.34214 |
| 3 | MGPTELLIEMEDWKGDK | 553.9429 | 8.527574 | 7 | 1.539432 |
| 3 | MKGLIDEVNQDFTNR | 504.5429 | 1.364865 | 7 | 0.270515 |
| 3 | MKPVPDLVPGNFK | 475 | 0.997497 | 5 | 0.209999 |
| 3 | MLTPEHVFIHPGWK | 490.4333 | 1.608312 | 6 | 0.327937 |
| 3 | MVETTAYALLTSLNLK | 541.9857 | 1.681694 | 7 | 0.310284 |
| 3 | NANFKFTDHLK | 452.22 | 2.429403 | 5 | 0.537217 |
| 3 | NKPGVYTDVAYYLAWIR | 580.0714 | 1.855365 | 7 | 0.319851 |
| 3 | NNKDSHSLTTNIMEILR | 521.9714 | 1.30603 | 7 | 0.250211 |
| 3 | NRDVVLTTTFVDDIK | 531.84 | 2.521507 | 5 | 0.47411 |
| 3 | NWGLSVYADKPETTK | 512.4 | 1.77426 | 6 | 0.346265 |
| 3 | PALPAGTEDTAKEDAANR | 533.7286 | 2.218644 | 7 | 0.415688 |
| 3 | PNSMVVEHPEFLK | 507.8 | 1.525341 | 7 | 0.300382 |
| 3 | RHPDYSVVLLLR | 494.6143 | 1.786457 | 7 | 0.361182 |
| 3 | RHPYFYAPELLFFAK | 523.8286 | 2.003925 | 7 | 0.382554 |
| 3 | RPYFPVAVGK | 464.72 | 1.903155 | 5 | 0.409527 |
| 3 | RQDNEILIFWSK | 494.22 | 2.775248 | 5 | 0.561541 |
| 3 | RRDGYLFQLLR | 471.6571 | 1.605052 | 7 | 0.3403 |
| 3 | RSFFSFLGEAFDGAR | 512.74 | 1.011435 | 5 | 0.197261 |
| 3 | RTHLPEVFLSK | 463.9857 | 2.054612 | 7 | 0.442818 |
| 3 | RTHLPEVFLSKVLEPTLK | 598.5571 | 1.674174 | 7 | 0.279702 |
| 3 | SEETKENEGFTVTAEGK | 492.4 | 2.397916 | 5 | 0.486985 |
| 3 | SELTQQLNALFQDK | 496.36 | 2.317973 | 5 | 0.466994 |
| 3 | SFFSFLGEAFDGAR | 497.74 | 1.529052 | 5 | 0.307199 |
| 3 | SGAQATWTELPWPHEK | 522.98 | 1.926655 | 5 | 0.368399 |
| 3 | SGFPQVSMFFTHTFPK | 535.5571 | 1.450123 | 7 | 0.270769 |
| 3 | SGKDPNHFRPAGLPEK | 491.48 | 1.89921 | 5 | 0.386427 |
| 3 | SGVQQLIQYYQDQK | 498.7333 | 2.577337 | 6 | 0.516777 |
| 3 | SHALQLNNR | 434.5833 | 2.159089 | 6 | 0.496818 |
| 3 | SIEVFGQFNGK | 448.52 | 2.130023 | 5 | 0.4749 |
| 3 | SKEFQLFSSPHGK | 479.1333 | 3.073543 | 6 | 0.64148 |
| 3 | SKEQLTPLIKK | 452.42 | 2.080144 | 5 | 0.459782 |
| 3 | SLAELGGHLDQQVEEFRR | 565.9857 | 1.910871 | 7 | 0.337618 |
| 3 | SPELQAEAKSYFEK | 524.2143 | 1.855109 | 7 | 0.353884 |
| 3 | SSLSVPYVIVPLK | 494.84 | 1.312631 | 5 | 0.265264 |
| 3 | SSNLIILEEHLK | 485.1667 | 1.94182 | 6 | 0.400238 |
| 3 | SYFEKSKEQLTPLIK | 521.9143 | 1.446177 | 7 | 0.277091 |
| 3 | SYFEKSKEQLTPLIKK | 540.0429 | 1.821956 | 7 | 0.337372 |
| 3 | SYFPESWLWEVHLVPR | 555.3143 | 1.576313 | 7 | 0.28386 |
| 3 | TAAQNLYEKTYLPAVDEK | 551.6571 | 1.725164 | 7 | 0.312724 |
| 3 | TAGWNIPMGLLYNK | 496.3 | 2.062765 | 5 | 0.415629 |
| 3 | TEHPFTVEEFVLPK | 501.1 | 1.739732 | 7 | 0.347183 |
| 3 | TEHYEEQIEAFK | 482.1429 | 1.664189 | 7 | 0.345165 |
| 3 | TELRPGETLNVNFLLR | 517.2286 | 1.7385 | 7 | 0.336118 |
| 3 | THLAPYSDELR | 452.22 | 1.143241 | 5 | 0.252806 |
| 3 | THLPEVFLSK | 441.55 | 1.960357 | 6 | 0.443972 |
| 3 | THLPEVFLSKVLEPTLK | 578.3571 | 1.58835 | 7 | 0.274631 |
| 3 | TLLPVSKPEIR | 483.7 | 1.793321 | 6 | 0.370751 |
| 3 | TMEQFTIHLTVNPQSK | 507.9571 | 1.328981 | 7 | 0.261633 |
| 3 | TMTIHNGMFFSTYDR | 492.6857 | 1.40051 | 7 | 0.28426 |
| 3 | TTIEKPVWLGFLGPIIK | 567.9714 | 2.139871 | 7 | 0.376757 |
| 3 | TTNIQGINLLFSSR | 500.6 | 2.147091 | 5 | 0.428904 |
| 3 | TVIGPDGHK | 406.1286 | 1.971222 | 7 | 0.485369 |
| 3 | TVMVNIENPEGIPVK | 561.04 | 1.840652 | 5 | 0.328079 |
| 3 | TWRNDLISATK | 451.9286 | 1.865221 | 7 | 0.412725 |
| 3 | TYLPAVDEKLR | 483.4 | 1.921805 | 7 | 0.39756 |
| 3 | TYLPAVDEKLRDLYSK | 539.7857 | 1.604606 | 7 | 0.297267 |
| 3 | VDVIPVNLPGEHGQR | 528.5286 | 1.762304 | 7 | 0.333436 |
| 3 | VEDPESTLFGSVIR | 500.78 | 2.804817 | 5 | 0.56009 |
| 3 | VFDEFKPLVEEPQNLIK | 533.2571 | 1.713532 | 7 | 0.321333 |
| 3 | VGPEADKYR | 421.9571 | 1.833809 | 7 | 0.434596 |
| 3 | VIGNMGQTMEQLTPELK | 529.0167 | 1.779232 | 6 | 0.336328 |
| 3 | VKDISEVVTPR | 451.6 | 2.124147 | 6 | 0.47036 |
| 3 | VKDLATVYVDVLK | 491.85 | 1.778482 | 6 | 0.36159 |
| 3 | VKSPELQAEAK | 452.76 | 2.943297 | 5 | 0.650079 |
| 3 | VKSPELQAEAKSYFEK | 530.5286 | 1.612156 | 7 | 0.303877 |
| 3 | VLSLAQEQVGGSPEK | 486.84 | 1.683152 | 5 | 0.34573 |
| 3 | VMDKYTFELSR | 467.3143 | 1.258873 | 7 | 0.269385 |
| 3 | VPEARPNSMVVEHPEFLK | 556.9429 | 1.594634 | 7 | 0.286319 |
| 3 | VPGLYYFTYHASSR | 517.7143 | 1.218508 | 7 | 0.235363 |
| 3 | VPLLLSEPINIIDALEMR | 576.6429 | 1.217726 | 7 | 0.211175 |
| 3 | VPQVSTPTLVEVSR | 475.08 | 1.706458 | 5 | 0.359194 |
| 3 | VQFELHYQEVK | 494.22 | 1.7936 | 5 | 0.362915 |
| 3 | VRGGEGTGYFVDFSVR | 494.5857 | 1.439246 | 7 | 0.291 |
| 3 | VTIMWTPPESAVTGYR | 533.34 | 1.40819 | 5 | 0.264032 |
| 3 | VTWAPPPSIDLTNFLVR | 570.7571 | 2.204433 | 7 | 0.38623 |
| 3 | VVLHPNYSQVDIGLIK | 548.2143 | 1.915227 | 7 | 0.349357 |
| 3 | WFYIASAFRNEEYNK | 513.02 | 1.741264 | 5 | 0.339414 |
| 3 | WKNFPSPVDAAFR | 467.4429 | 2.000714 | 7 | 0.428013 |
| 3 | WYEIEKIPTTFENGR | 503.8714 | 1.831861 | 7 | 0.363557 |
| 3 | YEASILTHDSSIR | 482.2833 | 1.991398 | 6 | 0.41291 |
| 3 | YEITTIHNLFR | 477.66 | 1.388524 | 5 | 0.290693 |
| 3 | YKEENDDFASFRVDR | 521.1333 | 2.288813 | 6 | 0.439199 |
| 3 | YLQEIYNSNNQKIVNLK | 522.9857 | 1.860491 | 7 | 0.355744 |
| 3 | YNPVVIDFEMQPIHEVLR | 545.9571 | 1.443211 | 7 | 0.264345 |
| 3 | YVGGQEHFAHLLILR | 521.6667 | 1.98964 | 6 | 0.381401 |
| 3 | YVGGQEHFAHLLILRDTK | 576.3 | 1.451436 | 7 | 0.251854 |
| 3 | YVNKEIQNAVNGVK | 496.1 | 2.120535 | 7 | 0.427441 |
| 4 | DLMEKVKSPELQAEAK | 618.5667 | 2.839484 | 6 | 0.459042 |
| 4 | DSQEEEKTEALTSAKR | 652.2167 | 3.413747 | 6 | 0.523407 |
| 4 | ENADSLQASLRPHADELK | 668.1429 | 1.66519 | 7 | 0.249227 |
| 4 | EPAHLMSLFGGKPMIIYK | 697.7571 | 2.114125 | 7 | 0.302989 |
| 4 | ERGHMLENHVER | 601.3429 | 1.977252 | 7 | 0.328806 |
| 4 | FSYSSGHVHLSSENK | 605.18 | 2.284075 | 5 | 0.377421 |
| 4 | FTVDRPFLFLIYEHR | 645.0857 | 2.280664 | 7 | 0.353544 |
| 4 | GETHEQVHSILHFK | 589.6714 | 1.958498 | 7 | 0.332134 |
| 4 | LGPHAGDVEGHLSFLEK | 640.7714 | 2.536871 | 7 | 0.395909 |
| 4 | LKEEIGKELEELR | 621.5167 | 2.897873 | 6 | 0.466258 |
| 4 | LQHLENELTHDIITK | 625.9857 | 2.56478 | 7 | 0.409719 |
| 4 | LRTEGDGVYTLNDKK | 613.5 | 2.099206 | 7 | 0.342169 |
| 4 | LSINTHPSQKPLSITVR | 663.0857 | 2.440921 | 7 | 0.368115 |
| 4 | LTIGEGQQHHLGGAK | 604.56 | 2.577402 | 5 | 0.426327 |
| 4 | MLTPEHVFIHPGWK | 644.5 | 1.949359 | 6 | 0.302461 |
| 4 | NNKDSHSLTTNIMEILR | 655.86 | 1.517564 | 5 | 0.231385 |
| 4 | PPEIAHGYVEHSVR | 613.8167 | 2.594931 | 6 | 0.422753 |
| 4 | RHPYFYAPELLFFAK | 643.72 | 1.875367 | 5 | 0.291333 |
| 4 | RTHLPEVFLSKVLEPTLK | 675.9571 | 1.630805 | 7 | 0.241259 |
| 4 | SKEFQLFSSPHGK | 584.6167 | 3.706436 | 6 | 0.633994 |
| 4 | SLAELGGHLDQQVEEFRR | 661.9143 | 2.365325 | 7 | 0.357346 |
| 4 | SYFEKSKEQLTPLIK | 648.5167 | 2.011384 | 6 | 0.310152 |
| 4 | SYFEKSKEQLTPLIKK | 614.15 | 2.697962 | 6 | 0.4393 |
| 4 | THLPEVFLSKVLEPTLK | 686.1429 | 2.473767 | 7 | 0.360532 |
| 4 | TYLPAVDEKLRDLYSK | 634.4 | 2.439945 | 7 | 0.384607 |
| 4 | VKSPELQAEAKSYFEK | 655.7286 | 2.750584 | 7 | 0.41947 |
| 4 | VYSLNDDLKPAK | 575.9714 | 3.380194 | 7 | 0.586868 |
| 4 | YKEENDDFASFRVDR | 643.2 | 2.990541 | 7 | 0.464947 |
| 4 | YVGGQEHFAHLLILR | 653.3571 | 2.368443 | 7 | 0.362504 |
